# Translating Evidence for Early Intervention in Psychosis (TRANSLATE): Protocol for Evaluating the Implementation of Early Intervention in Psychosis in Low and Lower‐Middle Income Countries (LMICs)

**DOI:** 10.1111/eip.70243

**Published:** 2026-10-21

**Authors:** Saeed Farooq, Nishani Fonseka, Nalinda Wellappuli, Abbie Milner, Saima Sheikh, Abdul Jalil Khan, Mian Mukhtar‐ul‐Haq Azeemi, Muhammad Firaz Khan, Laila Noushad, Imtiaz Ahmad Dogar, Aftab Alam Khan, Hazrat Ali Khan, Moin Ahmed Ansari, Athula Sumathipala, Rumi Ruben, Ivonne Solis‐Trapala

**Affiliations:** ^1^ Faculty of Medicine and Health Sciences Keele University Newcastle‐under‐Lyme UK; ^2^ Rheumatology and Musculoskeletal Sciences, Nuffield Department of Orthopaedics University of Oxford Oxford UK; ^3^ Institute of Family Medicine, Khyber Medical University Peshawar Pakistan; ^4^ Psychiatry Department Lady Reading Hospital Peshawar Pakistan; ^5^ Institute of Public Mental Health & Behavioural Sciences, Khyber Medical University Peshawar Pakistan; ^6^ Department & Organization Office of Research, Innovation, and Commercialization (ORIC) Khyber Medical University Peshawar Peshawar Pakistan; ^7^ Department of Psychiatry and Behavioural Sciences Faisalabad Medical University Faisalabad Pakistan; ^8^ Department of Psychiatry and Behavioural Sciences Ayub Medical Teaching Institute Abbottabad Pakistan; ^9^ Balochistan, Institute of Psychiatry and Behavioural Science Quetta Pakistan; ^10^ Medicine and Allied Specialties Liaquat University of Medical and Health Sciences Jamshoro Pakistan; ^11^ Institute for Research and Development in Health and Social Care Battaramulla Sri Lanka; ^12^ Karapitiya Teaching Hospital Galle Sri Lanka

**Keywords:** early intervention in psychosis, evidence and policy, first episode psychosis, implementation research, prognostic model, treatment resistant schizophrenia

## Abstract

**Background:**

In high‐income countries, early intervention in psychosis (EIP) services provide treatment for first episode psychosis (FEP) as early as possible, proven to effectively improve health outcomes. These services rarely exist in low‐ and lower‐middle‐income countries (LMICs). The TRANSLATE study aims to evaluate the implementation of the EIP services using the selected indicators and to develop a clinical tool to predict the risk of Treatment‐Resistant Schizophrenia (TRS) in patients with FEP.

**Methods:**

This is an observational implementation research study conducted across five sites in Pakistan and one in Sri Lanka, recruiting 670 FEP patients. The evaluation of the implementation of the service will include key clinical and process outcomes such as disengagement from services, rates of remission, the level of functioning, quality of life, psychopathology and physical health. Required data will be collected over a 1‐year period at baseline and at 1, 3, 6 and 12‐month intervals. A descriptive analysis of baseline data and service utilisation data will be conducted, stratified by site. Probabilities of disengagement, remission and process evaluation outcomes will be modelled using logistic regression models adjusting for duration of untreated psychosis, baseline symptom severity and geographical location. Selected key clinical outcomes will also be analysed and presented descriptively. To develop a clinical model prediction model for TRS, a penalised multivariable logistic regression model will be fitted to estimate the one‐year risk of TRS following an FEP diagnosis, incorporating up to 15 predictor variables identified within the study cohort. Model performance will be assessed using calibration plots and the C‐statistics for discrimination. Internal validation will be carried out by bootstrapping and the optimism‐adjusted calibration slope will be used as a global shrinkage factor to account for overfitting, with the model intercept re‐estimated to ensure calibration‐in‐the‐large.

**Discussion:**

This will be the first large study that will evaluate the implementation of EIP service in a LMIC country setting. The findings of this study will inform the adoption and scaling‐up of EIP services in routine clinical practice. The clinical prediction model for the early detection of TRS will help initiate effective treatments for TRS at the earliest possible stage and potentially reduce the significant burden of disease associated with TRS.

**Trial Registration:** ClinicalTrials.gov identifier: ISRCTN16495294.

**Abbreviations**
CBT‐p: cognitive behavioural therapy for psychosis
CEI: community engagement and involvement
EIP: early intervention in psychosis
FEP: first episode psychosis
FIM: feasibility of intervention measure
HICs: high‐income countries
IAM: intervention appropriateness measure
LMICs: low‐ and lower‐middle‐income countries
NICE: National Institute for Health and Care Excellence
PANSS: positive and negative syndrome scale
PPIE: patient and public involvement and engagement
RAs: research assistants
TRANSLATE: translating evidence for early intervention psychosis
TRRIP: treatment response and resistance in psychosis
TRS: treatment‐resistant schizophrenia
WPs: work packages
YMRS: Young Mania Rating Scale

## 1 Background

First episode psychosis (FEP) typically refers to the first ever psychotic episode in people who either have not received any antipsychotics or have used medication for no longer than 6 weeks (Breitborde et al. 2009). FEP is a stage of severe mental illness characterised by significant changes in perception, thought processes, mood and behaviour, often accompanied by positive symptoms like hallucinations and delusions and negative symptoms like emotional apathy and social withdrawal. Anxiety, depression and co‐existing substance misuse are also commonly present (National Institute for Health and Care Excellence (NICE) 2016).

Untreated or partially treated FEP disrupts education, reduces long‐term employment, is associated with a huge economic burden and is significantly higher in mortality than the general population (Farooq et al. 2009). Approximately 10% of individuals experience self‐harm during extended periods of untreated psychosis (Lora et al. 2012). The duration of untreated psychosis (DUP), defined as the period between the onset of psychosis and initiating antipsychotic treatment, is 125 weeks in low‐ and middle‐income countries (LMICs) compared to 62.5 weeks in high‐income countries (HICs) (Large et al. 2008). Even when treatment is initiated, it is mostly limited to acute episodes. Almost 70% of people with psychosis in LMICs do not receive regular care, resulting in a huge treatment gap (De Silva et al. 2014).

There is overwhelming evidence that deterioration occurs aggressively in the first 2 to 5 years of early psychosis and it is crucial to intervene in this period (Birchwood et al. 1998; Coentre et al. 2011). This has led to the development of early intervention for psychosis (EIP) programmes in HICs. EIP services provide treatment at the earliest possible time after diagnosis of FEP (McDaid et al. 2016; Fusar‐Poli et al. 2017). Systematic reviews and meta‐analyses have shown that EIP services result in significantly improved clinical, social and vocational outcomes. This includes reductions in psychiatric hospitalisations, symptom severity and improvements in personal and social functioning, with more patients returning to employment or education (Correll et al. 2018; Farooq et al. 2023; Pipkin 2021). A longitudinal observational study conducted in England demonstrated that patients in EIP services were 116% more likely to gain employment (Tsiachristas et al. 2016). Furthermore, improved outcomes are demonstrated across different settings and models of early intervention (Correll et al. 2018; Fusar‐Poli et al. 2017; Pipkin 2021). A recent meta‐analysis also showed that EIP services are cost‐effective (Aceituno et al. 2019).

However, none of the systematic reviews or other evidence evaluating EIP programmes included a study from an LMIC. This represents a huge gap in youth mental health care, considering that almost 90% of 1.8 billion young people aged 10 to 24 years—the highest risk period for early psychosis—live in these countries (UNFPA 2014). Despite limited evidence from a few countries, Farooq et al. found that low‐ and middle‐income countries (LMIC) can establish and adapt the key elements of early intervention in psychosis (EIP) in resource‐limited settings (Farooq, Fonseka, et al. 2024). EIP models proven effective in the HIC settings cannot be directly translocated to LMIC settings. The development and implementation of an EIP model, which consists of these elements, need to be culturally adapted and acceptable to ensure that it fits local needs effectively (Singh and Javed 2020). Strategies such as involving the Traditional Spiritual Healers (TSH) in the care pathway, the engagement of non‐specialist health care personnel and active involvement of family members need to be considered (Farooq, Fonseka, et al. 2024; Iyer et al. 2022).

### 1.1 Treatment‐Resistant Schizophrenia (TRS) and First Episode Psychosis

First Episode Psychosis includes several psychotic disorders, but it is estimated that about 70% of individuals with FEP have a diagnosis of schizophrenia (De Silva et al. 2014). It is estimated that almost one‐third of people with schizophrenia develop into treatment‐resistant schizophrenia (TRS). TRS is defined as failure to respond to two sequential courses of antipsychotic treatment.

Patients with TRS have higher unemployment rates, worse quality of life and poorer social and occupational functioning than those who respond to treatment (Kennedy et al. 2014).

Systematic review evidence indicates that early clozapine use may improve outcomes in first‐episode schizophrenia patients (Okhuijsen‐Pfeifer et al. 2020). Our systematic review revealed that poor adherence by the patients, lack of awareness and difficulty in identifying TRS in the early stages resulted in long delays and underutilisation of clozapine (Farooq et al. 2019).

A systematic review published in 2022 identified early prediction models for FEP in the literature, including two studies that specifically aimed to predict TRS (Lee et al. 2022).

In a previous research study, we developed and internally validated a clinical prediction model for the risk of treatment‐resistant schizophrenia in the first episode of psychosis (Farooq, Hattle, et al. 2024). This model (SPIRIT prediction tool) included seven clinical predictors (e.g., age, DUP, substance abuse, premorbid adjustment) derived from two UK cohorts (Aetiology and Ethnicity in Schizophrenia and Other Psychoses—AESOP‐10 and Genetic and Psychosis—GAP) of FEP patients. The major limitations of the SPIRIT tool were the use of only seven predictor variables, a retrospective cohort study design with missing data and the limited generalisability of data from two UK urban sites.

### 1.2 Development of Early Intervention in Psychosis Services

Prior to evaluating the implementation of EIP services, the research team developed and adapted the EIP service model for these two countries. There is no definitive EIP model for individuals with FEP and the general mental health system provides these services in Pakistan and Sri Lanka. We followed an iterative process that included evidence synthesis, consultation with all stakeholders and consensus‐building to develop a culturally acceptable EIP service model for managing individuals experiencing FEP in a hospital setting. We conducted a systematic review of EIP and the management of FEP in LMICs (Farooq, Fonseka, et al. 2024). A feasibility study was conducted in Pakistan, known as THEHOPE (Farooq et al. 2023), to examine the referral care pathway from TSH to primary care physicians for suspected FEP individuals. We conducted focus group discussions (FGDs) (*n* = 15) at the research sites in Pakistan (*n* = 9) and Sri Lanka (*n* = 6), involving service users, physicians, psychiatrists, health administrators and traditional spiritual healers to gather stakeholders' perspectives. In addition, five in‐depth interviews were conducted in the UK with mental health professionals who have experience with mental health services in Pakistan and the EIP model in the UK.

Qualitative interviews were followed by two theories‐of‐change workshops that mapped the resources, processes and implementation strategies for implementing the EIP service model. Finally, two rounds of consensus‐building workshops using the nominal group technique were held to reach consensus on the draft EIP model. The main service components of the EIP model include pharmacological management, psychosocial interventions, which include family intervention and cognitive behavioural therapy for psychosis (CBT‐p) and physical health monitoring.

This article is focused mainly on the quantitative aspects of the evaluation of EIS and development of the prognostic model for predicting treatment‐resistant schizophrenia (see below). The details on the development and cultural adaptation of the EIP service model are provided in Additional File S1, details of other work packages of the study provided in Additional File S2.

### 1.3 Study Aims

The Translating Evidence for Early Intervention Psychosis (TRANSLATE) programme of research aims to evaluate the implementation of EIP services, and to develop and validate a prognostic model for early identification of patients who could be at risk of TRS in patients presenting with first episode of psychosis. The implementation and evaluation will be carried out in two LMICs (Pakistan and Sri Lanka) using key selected indicators. The TRANSLATE study will also develop a clinical tool to predict the 1‐year risk of TRS in patients with FEP, using data from a cohort of study participants within the EIP programme. The development and validation of an improved prediction model for TRS in FEP (SPIRIT +) will include a larger number of potential predictive variables derived from the prospective cohort recruited in the TRANSLATE study and will overcome the limitations of previous studies.

## 2 Methods

### 2.1 Study Setting

The TRANSLATE study will be set across five study sites in Pakistan and one in Sri Lanka. Pakistan has a population of over 250 million and five provinces. Each of the five provinces in Pakistan will have a study site to ensure a good representation of urban and rural populations and all ethnic groups. We will conduct this study at one site in the southern province of Sri Lanka.

### 2.2 Study Design

We will use an observational implementation design, which is also termed post‐design in the implementation science literature (Knox et al. 2023). The post‐design examines healthcare processes and healthcare utilisation or output after the introduction of an evidence‐based clinical intervention in a health setting. We will recruit FEP participants for the study and assemble a cohort. The data from the study cohort will be utilised to evaluate the implementation of EIP services and to develop a prediction model for early identification of TRS patients.

### 2.3 Study Population and Recruitment

Study participants will be recruited from 6 study sites and engagement activities with stakeholders and communities will be conducted to facilitate early referral of study participants to recruitment sites. The patient flow is described in Figure 1. All consecutive patients presenting to the selected study sites will be enrolled according to the study's prescribing protocol and inclusion/exclusion criteria.

**FIGURE 1:**
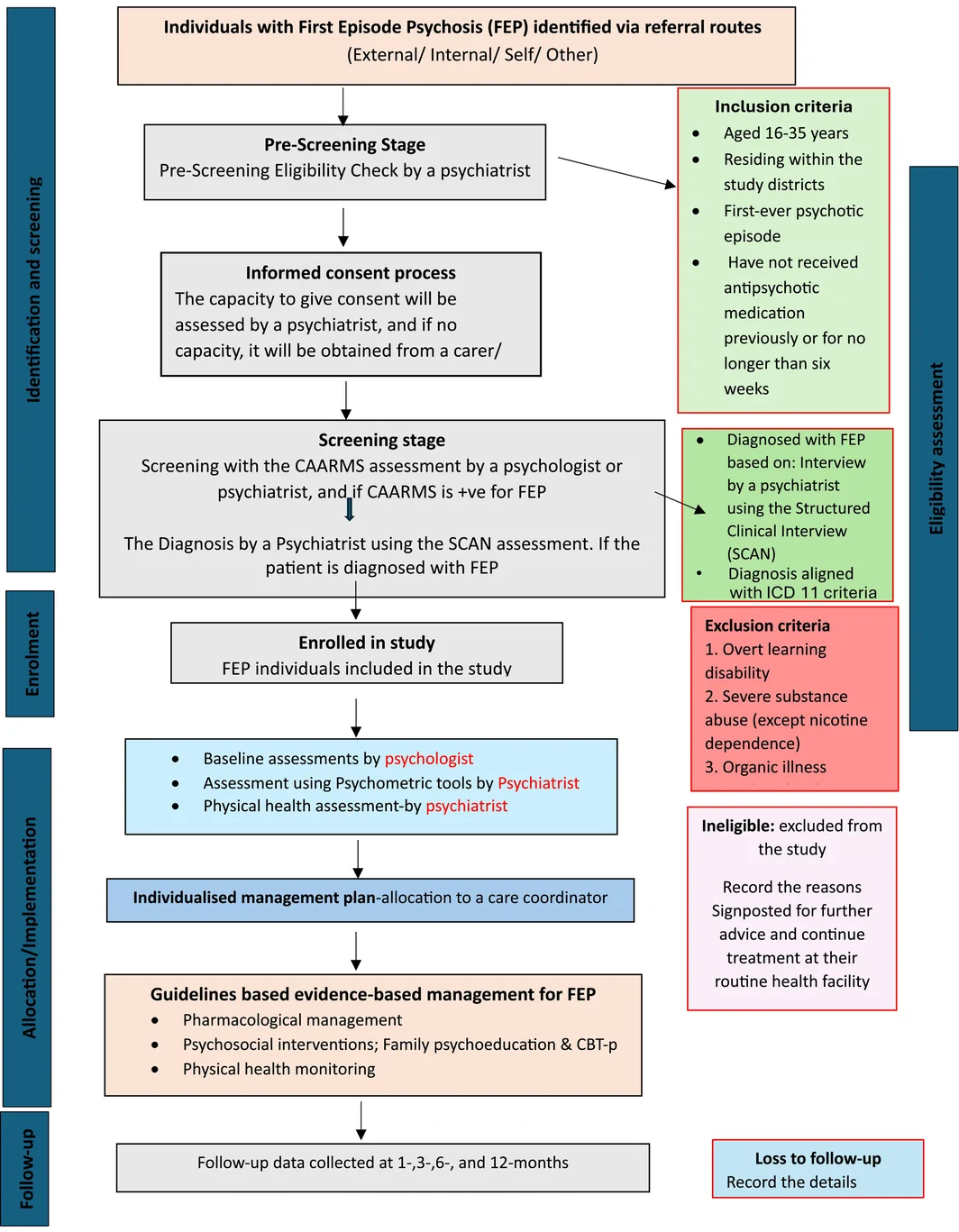
TRANSLATE research study key processors.

### 2.4 Aim 1: Evaluation of the Implementation of EIP Services

We have identified the following indicators to evaluate the implementation of EIP services across the six study sites. The study team selected disengagement of service and remission as composite indicators to evaluate mental health service delivery. Additional key clinical indicators were selected to further describe the health outcomes. In line with the implementation science literature, we will be conducting the process evaluation defined by Proctor's framework (Proctor et al. 2011).

#### 2.4.1 Disengagement From the Service

The TRANSLATE study will estimate the proportion of patients who have disengaged from the service as the primary evaluation outcome. Disengagement from services is considered one of the most important outcomes of EIP services and is used widely in EIP services (NHS England, National Collaborating Centre for Mental Health, and National Institute for Health and Care Excellence 2016; Tait et al. 2003). Disengagement from services will be defined as a participant's refusal to engage with an EIP service or not responding to EIP contact requests for a consecutive 3‐month period during follow‐up, while still residing within the study site catchment area (NHS England et al. 2016; Tait et al. 2003).

#### 2.4.2 Remission

The remission from FEP measures the quality of service in providing effective treatment (Lally et al. 2017). The definition of remission in schizophrenia and bipolar affective disorders, the two most common categories in FEP, varies considerably. Most studies adopt a criterion of symptom resolution and a minimum duration of 1–6 months, during which the symptoms are below a certain cut‐off point on different rating scales (van Os et al. 2006). For this study, remission will be defined as the absence of overt psychotic symptoms (operationalised as a score of 3 or below i.e., mild or lower on eight core psychotic symptoms on the Positive and Negative Syndrome Scale (PANSS)) (Kay et al. 1987) or a total score ≤ 12 for bipolar disorders measured by the Young Mania Rating Scale (YMRS) that is maintained for at least 1 month (Young et al. 1978).

#### 2.4.3 Key Clinical Outcomes

We will also record key clinical outcomes to examine the effect of EIP services on improvement in symptoms and functioning, family burden, perceived stigma, relapse and physical health. The relapse will be defined as one of the following: exacerbation/re‐emergence of symptoms requiring hospital admission, or the patient no longer meeting the criteria for remission, change of medication or patient management, deliberate self‐harm or violent behaviour, or significant suicidal or homicidal ideation (Olivares et al. 2013).

The relapse, remission and disengagement from services will be carefully monitored and recorded by care coordinators during the follow‐up period. The care coordinators will contact participants at monthly intervals to monitor these outcomes and treatment progress. All the other key clinical outcomes will be measured at baseline and at 1‐, 3‐, 6‐ and 12‐month follow‐up. The assessment tools that will be used to measure the other key clinical outcomes with the follow‐up points are described in Table 1.

**TABLE 1:** All instruments to be used in the TRANSLATE study across time points.

Description	Measure	Screening assessment	Baseline assessment	1 month assessment	3 months assessment	6 months assessment	12 months assessment
Age	Date of birth	✓					
Gender	Male/female	✓					
Address		✓					
Education	Years spent in education	✓					
Marital status		✓					
Employment status		✓					
Screening for FEP	Comprehensive Assessment of At‐Risk Mental States (CAARMS)	✓					
Sociodemographic factors	MRC Sociodemographic Schedule modified	✓					
Psychopathology/symptom severity^a^	Positive & Negative Syndrome Scale (PANSS) Young Mania Rating Scale (YMRS) Hamilton Depression Rating Scale (HDRS)		✓	✓	✓	✓	✓
Overall illness severity and improvement^a^	Clinical Global Impression Scale (CGI)		✓	✓	✓	✓	✓
Screening/other instruments	WHO STEPS—modified	✓					✓
Substance abuse	Drug abuse screening tool (DAST)		✓	✓	✓	✓	✓
Side effects^a^	Glasgow Antipsychotics Side Effects Scale (GAS)		✓	✓	✓	✓	✓
Baseline motor function	Abnormal Involuntary movement scale (AIMS)		✓	✓	✓	✓	✓
Adherence to antipsychotic treatment	Medicine Adherence Rating Scale (MARS)			✓	✓	✓	✓
Occupational and social functioning	WHO Disability Assessment Schedule Version 2 (WHODAS 2.0)		✓	✓	✓	✓	✓
Quality of life	EuroQol‐5		✓	✓	✓	✓	✓
Perceived stigma^a^	Internalised Stigma of Mental Illness Inventory	✓					✓
Family burden^a^	Family Burden Scale	✓			✓		✓
Cognition	Montreal Cognitive Assessment (MoCA)	✓	✓	✓	✓	✓	✓
Physical health measurements	Blood pressure, Body Mass Index, waist circumference	✓	✓	✓	✓	✓	✓

^a^ Key clinical outcomes.

#### 2.4.4 The Implementation Process Evaluation

The process of implementation of the EIP service will be evaluated using both qualitative and quantitative methods. These implementation outcomes for the process evaluation will be guided by the domains of the Proctor's framework (Proctor et al. 2011).

##### 2.4.4.1 Quantitative Methods in Process Evaluation

Service utilisation metrics gathered during setting up and providing the EIP will be used for measuring acceptability, adoption, appropriateness, feasibility, fidelity, implementation costs, penetration and sustainability. Acceptability is defined as the tolerability or satisfaction of the approach being used, while feasibility is the utility of the intervention or suitability and practicability for the setting in which it is implemented (Proctor et al. 2011). These will be measured using service utilisation data, for example, patients screened for suspected psychosis out of all those presenting to services, the median time taken in screening, proportion of adherence to treatment procedures by age group, frequency and duration of contacts, median length of time to treatment from assessment to starting treatment and proportion of cases identified as At‐Risk Mental State (ARMS) for FEP out of total accepted cases.

The Intervention Appropriateness Measure (IAM) and Feasibility of Intervention Measure (FIM) (Weiner et al. 2017) will be completed by those delivering psychiatric services, including doctors, nurses and psychologists.

To measure the fidelity of the intervention, the First Episode Psychosis Services Fidelity Scale will be modified and adapted based on standards for our service (Addington 2021). This will assess the degree to which mental health teams deliver evidence‐based care for FEP. We will follow the clinical practice guielines developed for Early Psychosis management in Pakistan (Farooq and Milner 2024).

##### 2.4.4.2 Qualitative Methods in Process Evaluation

Qualitative methods will be used to explore experiences of the intervention, assess the perceptions of acceptability, feasibility, appropriateness and adoption of the intervention, as well as barriers and facilitators for future scale‐up. We aim to conduct up to five FGDs, involving six to eight participants in each group, in sites in Pakistan (one in each province) and Sri Lanka. FGDs will include key stakeholders involved in the delivery or use of the EIP, such as healthcare workers and service users.

We will also conduct semi‐structured interviews (*n* = 20) with stakeholders in the wider healthcare system, including health planners, policymakers and administrators, to understand future scale‐up challenges. Data collection for FGDs and interviews will follow topic guides informed by Proctor's model for implementation outcomes and relevant theories of intervention acceptability (Proctor et al. 2011). A deductive but flexible framework will guide analysis informed by underpinning theory; data collection and analysis will be iterative and end once saturation has been achieved (Saunders et al. 2018).

##### 2.4.4.3 Implementation Costs

The Client Service Receipt Inventory will measure direct and indirect costs in the programme to estimate health economic costs from the payer and provider perspectives (Beecham and Knapp 2001). We will estimate mean, median and quartile costs at each time point. We will also conduct a bottom‐up costing of the typical package of care offered to those participating in the programme (Doyle et al. 2014; Lally et al. 2017; Riley et al. 2020).

### 2.5 Participant Eligibility Criteria

#### 2.5.1 Inclusion Criteria

We will recruit all patients aged 16–40 years and residing within the study districts experiencing a first‐ever psychotic episode who have not received antipsychotic medication previously, or if they have already used antipsychotic medications, it was for no longer than 6 weeks (Breitborde et al. 2009). The FEP diagnosis will be made via interview by psychiatrists using Schedules for Clinical Assessment in Neuropsychiatry (SCAN) and will be based on the International Classification of Diseases, 11th revision (ICD‐11) criteria for schizophrenia, persistent delusional disorder, acute and transient psychotic disorders, schizoaffective disorder, mood disorders (mania, severe depressive episode, bipolar affective disorder) with psychotic symptoms. We will also include participants with severe substance abuse.

#### 2.5.2 Exclusion Criteria

We will exclude the participants who have received and used antipsychotic treatment continuously for a period longer than 6 weeks, or those with overt learning disabilities. We will exclude individuals with nicotine dependence or those with organic illness associated with psychotic illness.

### 2.6 Follow‐Up

All patients registered in the EIP service will receive a package of interventions which includes pharmacological management, psychosocial interventions and physical health monitoring for 1 year, following the standard guidelines for managing FEP as outlined above. Trained research assistants (RAs) will collect data on implementational, clinical and economic outcomes using standardised tools at baseline and follow‐up assessments in 1, 3, 6 and 12 months.

### 2.7 Aim: 2. Development of a Clinical Tool to Predict the Risk of Treatment‐Resistant Schizophrenia

The development of the SPIRIT+ will be guided by the protocol we developed and published for developing the SPIRIT model (Farooq et al. 2022). We will follow the prognosis research strategy (PROGRESS) framework (Hemingway et al. 2013; Riley et al. 2013; Steyerberg et al. 2013) and will report adhering to the transparent reporting of a multivariable prediction model of individual prognosis or diagnosis (TRIPOD) guidelines (Collins et al. 2015).

A nested study within the cohort of FEP patients of the EIP programme described above will be used to develop and internally validate the SPIRIT+ tool. All patients consenting to participate in EIP will form the cohort for developing and validating the SPIRIT+ clinical tool. For this study, the primary outcome measure will be the occurrence of TRS during the follow‐up period for predicting the risk of TRS within 1 year of starting treatment. TRS will be defined in line with the Treatment Response and Resistance in Psychosis (TRRIP) working group consensus guidelines (Howes et al. 2017). The TRRIP guidance recommends that TRS is defined as failure to respond to prior treatment consisting of ≥ 2 different antipsychotic trials, each for a minimum duration (≥ 6 weeks at a therapeutic dose) and dose (equivalent of Chlorpromazine 600 mg per day) despite the evidence of good treatment adherence (Farooq et al. 2022; Kennedy et al. 2014).

Response and remission will be measured using a standardised rating scale as described above. Similar to earlier work conducted in the same geographical regions (Morgan et al. 2014; Revier et al. 2015) and in keeping with work from Andreasen et al. (2005) remission is defined as the absence of overt psychotic symptoms (operationalised as a score of 2 or 3 on rating scale 2 in the SCAN13; 0 = absence, 1 = symptom occurred, but fleeting, 2 = symptom definitely present, 3 = symptom present more or less continuously) for 6 months or longer. This definition of remission is not dependent on the presence of non‐psychotic symptoms (e.g., depressed mood), nor on whether the patients were receiving treatment during remission (Collins et al. 2015; Farooq et al. 2022; Hemingway et al. 2013; Riley et al. 2013; Steyerberg et al. 2013).

As part of the SPIRIT study (Farooq et al. 2022; Farooq, Hattle, et al. 2024), we identified a list of 22 candidate predictors of TRS from an extensive literature search and expert consultations (Farooq, Hattle, et al. 2024) and we used only seven predictors to develop the prediction model due to the limitation of the cohorts used for developing the model. In this study, we will collect data on a large number of clinical and demographic variables (see Table 1) and our sample size consideration allows us to use 15 predictor parameters. We will collect the data for these potential predictor variables at baseline and follow‐up periods at 3, 6 and 12 months and will use these to predict treatment resistance.

The prediction model will estimate the probability (risk) of TRS for individual patients, which will be of importance to clinicians, patients and their care providers, as it will provide information on prognosis and can inform discussions regarding different treatment options.

### 2.8 Sample Size

Meta‐analyses estimate the rates of disengagement from EIP services of about 15%–30% and remission rates of about 71% from FEP studies in Asia (Doyle et al. 2014; Lally et al. 2017). For the primary implementation outcomes, a sample size of 536 will allow estimation of the proportions of disengagement and remission with a margin of error of 0.04, assuming true proportions of 0.30 and 0.71, respectively, using two‐sided 95% Wilson score confidence intervals.

For the prediction model development (see below), assuming a Cox‐Snell R‐squared of 0.22 from our SPIRIT study, a minimum sample size of 536 (Riley et al. 2020) would allow consideration of 15 predictor parameters. The sample size also ensures that the overall outcome proportion will be estimated precisely, with upper and lower values of the 95% CI within a margin of 0.04 of an assumed proportion of TRS of 0.29. Based on the incidence of psychosis (about 26.6 per 100 000 person‐years) with an estimated population of over 10 million in all sites, we expect about 5200 cases of FEP in target districts over 2 years. With a conservative estimate of 50% of patients presenting to health facilities in target districts and about 70% consenting, we anticipate having about 700 cases for the prognostic and implementation outcome parameter estimates. We will aim to recruit 670 participants to allow for a potential 20% loss to follow‐up.

### 2.9 Statistical Analysis

We will conduct a descriptive analysis of baseline data and service utilisation metrics, overall and stratified by geographical location. To evaluate the implementation of EIP services, the probabilities of disengagement and remission will be modelled using logistic regression, adjusting for DUP, baseline symptom severity and geographical location. For selected key clinical outcomes, including rates of response, relapses, contact with the services and inpatient admissions, will be analysed descriptively, including means and 95% confidence intervals, overall and stratified by district. A mixed‐method approach will be utilised in conducting the process evaluation. We will use quantitative data described with percentages and qualitative themes that emerged from thematic analysis will be triangulated to complete the process evaluation analysis.

For the prognostic model, a penalised multivariable logistic regression model will be developed to predict the one‐year risk of TRS following an FEP diagnosis, considering the predictor parameters identified in the study. Potential non‐linear effects of continuous variables and plausible interaction terms will be assessed. Patterns of missing data will be described and multiple imputations will be used if a missing‐at‐random mechanism seems plausible. Model performance will be evaluated using calibration plots to assess calibration and the C‐statistic to assess discrimination. Internal validation will be carried out by bootstrapping the full development dataset, estimating optimism in model performance and producing optimism‐adjusted estimates for calibration parameters and discrimination of predicted TRS risk. The optimism‐adjusted calibration slope will be applied as a global shrinkage factor to penalise predictor effects for overfitting and the intercept term will be re‐estimated to ensure calibration‐in‐the‐large. Sensitivity analyses will be conducted to assess model stability.

### 2.10 Ethical Approvals

Ethical approval has been obtained from the Research Ethics Committees of Keele University, UK (reference: 2025‐1028‐1363), Khyber Medical University, Pakistan (KMU/IPHSS/Ethics/2025/TE/260), National Bioethics Committee for Research Pakistan (Ref: No. 4‐87/NBCR‐1259/24‐25/64) and University of Ruhuna, Sri Lanka (2025/P/035). Additionally, the protocol was also registered within the UK's Clinical Study Registry (ISRCTN16495294) as an observational study.

### 2.11 Community Engagement and Involvement (CEI)

The TRANSLATE study incorporates Community Engagement and Involvement (CEI) alongside Patient and Public Involvement and Engagement (PPIE) at every stage. All PPIE activities, training and participation in various CEI initiatives will employ culturally appropriate methods. Members of the Community and Research User Group (RUG) support the TRANSLATE research study and the feedback garnered from consultations with these groups is instrumental in shaping and implementing the research activities. All governance and management committees for the project include both service users and community members.

## 3 Discussion

The effectiveness of EIP is well established in HICs, but there is almost a complete lack of evidence for how well it can be adapted for resource‐limited settings. Research related to implementation research and cost‐effectiveness of EIP services for FEP is limited in LMICs. Although the components of the EIP service model, such as pharmacotherapy, family interventions and psychosocial interventions, are described in a few studies from LMICs (Farooq, Fonseka, et al. 2024) the implementation and evaluation of a model for EIP is lacking in the literature. This study will evaluate the newly rolled‐out EIP model using predetermined indicators, clinical outcomes and process evaluation. The study will also identify challenges and barriers in establishing these services in Pakistan and Sri Lanka and future scale‐up.

The overall research study has three major innovations. First, as part of the overall programme preceding the evaluation component of the study, we followed a rigorous scientific method to adopt and develop an EIP service model, which includes services and care pathways (please refer to Additional File S1 for details). Second, following the adoption of the service delivery model, we will use a large cohort of people with FEP and standardised diagnostic tools. The cohort will be followed at regular intervals to assess the key implementation outcomes, the rates of remission and engagement with the service over a one‐year follow‐up. As previous studies are retrospective or cross‐sectional in nature, the TRANSLATE study will address a major gap in the knowledge on rates of remission, engagement and number of clinical outcomes in two LMIC countries.

The development of a prognostic model using data from the prospective cohort study will be another innovation in applying this mental health research method in both countries, which has not been adopted thus far. The development of the SPIRIT+ tool will be a major innovation in identifying TRS patients in the early psychosis phase to reduce DUP.

### 3.1 Impact

We will be implementing the adapted clinical guidelines for FEP in a real‐world setting, with trained mental health professionals delivering evidence‐based care and skilled in using the electronic health record system. The results of this study and process evaluation will inform the adoption and scaling up of the EIP services and first steps in adopting the SPIRIT+ tool in everyday clinical practice. Evidence from HICs suggests that EIP can have an incremental cost‐effectiveness ratio of approximately $51 600 per Quality Adjusted Life Year (Sediqzadah et al. 2022). From a societal perspective, this translates into much fewer hospitalisations and more years of employment over the course of the remaining life expectancy of young FEP patients (Sediqzadah et al. 2022). In LMIC, the impacts would be much greater given the huge existing treatment gaps. In addition, the EIP programme will result in significant capacity building in mental health service provision and research. The psychiatrists, psychologists and other mental health professionals at all sites will be trained using the adopted EIP model, whereas experts in EIP service will train the master trainers in evidence‐based management of FEP who, in turn, will train several mental health professionals at each site. This will help to scale up similar services in other regions.

This study helps to provide evidence beyond cross‐sectional studies in exploring the differences in organisational variables across different sites. Developing potential services has a large impact on the timely delivery of essential healthcare services for people with mental health conditions and it will have a wider socio‐economic impact for immediate family members and for the countries in developing a healthier generation.

### 3.2 Strengths and Limitations of the Proposed Study

The proposed study builds on our established collaborations with our research partners across Pakistan and Sri Lanka. We have developed competent PPIE groups in both countries to review our work throughout the research phase. The EIP service model has been developed with extensive theoretical and practical collaboration with the professionals who are providing services at the field level of both countries. Therefore, we believe the EIP model has been appropriately applied in both countries before recruiting participants for the follow‐up study.

In this study, we will be recruiting a large sample size, considering the prevalence of the disease burden. As described above, we will follow up with the study participants for a one‐year period with intermittent assessments, which provide opportunities to assess disease progression of the individual participants together with the service implementation. We will be using a mix of quantitative indicators and a mixed‐methods approach in process evaluation to evaluate the implementation of EIP services in real‐world settings and understand its strengths and limitations.

A major limitation of the study is the lack of a control group. We have adapted our EIP model from the well‐established model of EIP that has shown effectiveness in randomised controlled trials (Aceituno et al. 2019; Correll et al. 2018; Fusar‐Poli et al. 2017), but it is possible that the adapted model does not have the same effectiveness as those tested in previous RCTs. The assessor doing the follow‐up assessments will be part of the team that is providing EIP and hence, bias may be introduced in assessments of outcomes of the study. However, we have utilised standardised tools and training provided to each study stakeholder in assessing the health status within the follow‐up period to minimise any observation bias. Additionally, bias may be introduced by loss to follow‐up and patients may not adhere to the study procedures. It is well known that patients with poor prognostic factors such as negative symptoms, poor treatment adherence and lack of social skills may not engage the service well and are likely to disengage from the service, thus inflating the positive outcomes. Finally, the study sites are facing major risks in terms of political uncertainty and natural disasters, such as floods. This may increase the risk of loss to follow‐up of study participants and further increase the attrition bias.

## Funding

The TRANSLATE research programme is funded by the National Institute for Health and Care Research (NIHR), UK (grant number: NIHR 303164). The funding source has no role in the design of the research study, nor in the collection, analysis, or interpretation of data. Additionally, it has no role in the preparation or publication of the manuscript.

## Ethics Statement

Ethical approval has been obtained from the Research Ethics Committees of Keele University, UK (reference: 2025‐1028‐1363), Khyber Medical University, Pakistan (KMU/IPHSS/Ethics/2025/TE/260), National Bioethics Committee for Research Pakistan (Ref: No. 4‐87/NBCR‐1259/24‐25/64) and University of Ruhuna, Sri Lanka (2025/P/035). Before any interviews, both oral and written information about the study and its purpose will be provided in the local language. Potential participants will offer witnessed informed consent, either through a signature or thumbprint. If eligible participants are unable to sign the informed consent due to a lack of capacity, their legal guardians will be asked to provide consent. Once individuals have the capacity to understand the study procedures, they will be invited to provide their informed consent again. The capacity to give consent will be reassessed at each key data collection point of the study and consent will be re‐recorded. All methods will be carried out in accordance with relevant guidelines and regulations, including the Declaration of Helsinki.

## Consent

The authors have nothing to report.

## Conflicts of Interest

The authors declare no conflicts of interest.

## Supporting information


**File S1:** Protocol for Development of EIP model (WP‐1).

**File S2:** Diagram for the TRANSLATE Work Packages.

## Data Availability

Data sharing not applicable to this article as no datasets were generated or analysed during the current study.
