# Supplementary material for: Translating Evidence for Early Intervention in Psychosis (TRANSLATE): Protocol for Evaluating the Implementation of Early Intervention in Psychosis in Low and Lower‐Middle Income Countries (LMICs)
Source: Early Interv Psychiatry. 2026 Oct 21;20(10):e70243. doi: 10.1111/eip.70243 (PMC13686379; doi:10.1111/eip.70243)
Supplement: Supplementary file 2 [file EIP-20-0-s002.docx]

**Annexure 1- TRANSLATE PROTOCOL-WP 1**

**Protocol for the “Translating Evidence for Early Intervention in Psychosis (TRANSLATE) in Low and Lower-Middle Income Countries (LMIC): Implementation and Evaluation”**

**RESEARCH PROTOCOL**

Work Package One: Development of FEP guidelines and EIP service model

**Version 1.1: 29/07/2024**

Funder number: NIHR303164

Project short title: The TRANSLATE study

Table of Contents

[LIST OF ABBREVIATIONS	3](#_Toc173833861)

[KEY STUDY CONTACT	3](#_Toc173833862)

[STUDY SUMMARY	4](#_Toc173833863)

[1.	BACKGROUND	7](#_Toc173833864)

[1.1	Research Importance	7](#_Toc173833865)

[1.2	Review of existing evidence	8](#_Toc173833866)

[1.3	Stakeholder, patient, and public engagement	8](#_Toc173833867)

[1.4	Work Packages	9](#_Toc173833868)

[2	METHODS	10](#_Toc173833869)

[2.1	Study Design	10](#_Toc173833870)

[2.2	Study setting	10](#_Toc173833871)

[2.3	This protocol	10](#_Toc173833872)

[3.	WORK PACKAGE ONE – STAKEHOLDER ENGAGEMENT AND QUALITATIVE STUDIES TO UNDERSTAND THE IMPLEMENTATION CONTEXT (objective 1)	11](#_Toc173833873)

[3.1	Theory of Change (TOC) Workshops	12](#_Toc173833874)

[3.2	Qualitative study	12](#_Toc173833875)

[3.2.1 Focus group discussion	12](#_Toc173833876)

[3.2.2	In-depth interviews	13](#_Toc173833877)

[3.2.3	Data collection	14](#_Toc173833878)

[3.2.4	Data analysis	14](#_Toc173833879)

[3.3	Consensus-building workshops	14](#_Toc173833880)

[3.3.2	Stakeholder Engagement Workshop 1	15](#_Toc173833881)

[3.3.3	Stakeholder Engagement Workshop 2	15](#_Toc173833882)

[3.3.4	Stakeholder Engagement Workshop 3	15](#_Toc173833883)

[3.4	Cascade training	16](#_Toc173833884)

[3.5	Informed consent and ethical conduct	16](#_Toc173833885)

[3.5.2	Participant information sheet	16](#_Toc173833886)

[3.5.3	Protecting participants’ privacy and confidentiality	17](#_Toc173833887)

[3.5.4	Participant burden	18](#_Toc173833888)

[3.6	Data Management	18](#_Toc173833889)

[3.7	Patient and Public Involvement and Engagement	19](#_Toc173833890)

[3.8	Study Outcomes -WP 1	19](#_Toc173833891)

[3.9	Ethical and Regulatory Considerations	20](#_Toc173833892)

[3.9.2	Research Ethics Committee (REC) review & reports	20](#_Toc173833893)

[3.9.3	Peer review	20](#_Toc173833894)

[3.9.4	Access to the study dataset	20](#_Toc173833895)

# LIST OF ABBREVIATIONS

| **DUP** | Duration of untreated psychosis |
| --- | --- |
| **EIP** | Early interventions in psychosis |
| **FEP** | First Episode Psychosis |
| **FGD** | Focus Group Discussion |
| **HICs** | High-Income Countries |
| **IRD** | Institute for Research and Development |
| **KMU** | Khyber Medical University |
| **LMICs** | Low and middle-income countries |
| **NICE** | National Institute for Health and Care Excellence |
| **NGT** | Nominal Group Technique |
| **PPIE** | Patient and Public Involvement and Engagement |
| **SPIRIT** | Schizophrenia Prediction of Resistance to Treatment |
| **TRS** | Treatment-resistant schizophrenia |
| **TSH** | Traditional Spiritual Healers |
| **WP** | Work Package |
|  |  |

# KEY STUDY CONTACT

| Principal Investigator | Prof Saeed Farooq |
| --- | --- |
|  | Global Research Professor, National Institute of Health and Care Research, UK  Professor of Psychiatry and Public Mental Health, School of Medicine, Keele University  Honorary Consultant Psychiatrist, Midlands Partnership NHS Foundation Trust |
|  | [s.farooq@keele.ac.uk](mailto:s.farooq@keele.ac.uk) |
|  |  |
|  |  |
|  |  |
|  |  |

# STUDY SUMMARY

| **Study title** | Translating Evidence for Early Intervention in Psychosis (TRANSLATE) in Low and Lower-Middle Income Countries (LMIC): Implementation and Evaluation |
| --- | --- |
| **Short title** | The TRANSLATE study |
| **Study summary** | **Background:** Duration of Untreated Psychosis (DUP) (the period between the onset of psychosis and the initiation of antipsychotic treatment) is twice as long in LMICs compared to High-Income countries (HIC). Prolonged DUP is associated with poor prognosis, premature mortality, disrupted education, and poor employment prospects. Early Intervention in Psychosis (EIP) services are well established in HIC and provide treatment at the earliest possible time after diagnosis of first episode psychosis (FEP), resulting in significantly improved outcomes, but EIP hardly exists in LMIC. Treatment-resistant schizophrenia (TRS) is arguably the most disabling psychiatric disorder, but clozapine (the only licensed medication for TRS) gets delayed for several years, and there is no clinical tool available at present to identify TRS at the earliest possible stage in FEP. |
|  | **Aims and objectives:** The TRANSLATE (Translating evidence for the Early Intervention in Psychosis) programme aims to establish and evaluate EIP services in Pakistan and Sri Lanka and assemble a cohort of FEP within the EIP service for developing a clinical tool to predict the risk of TRS. |
|  | **Methods:** Mixed methods across the work package one and two. Stakeholder engagement and qualitative methods will be used to develop a model for EIP. A small pilot study will test the EIP model to identify implementation barriers and the use of guidelines in clinical practice. The EIP model will be implemented in six sites of Pakistan and Sri Lanka, using task-sharing and task-shifting approaches. The implementation and clinical outcomes will be evaluated using quantitative and qualitative methods. Using the same study population, a cohort will be formed to develop and validate the clinical tool for predicting the risk of TRS.  The work package three-capacity building will run through the course of the study. |
|  | **Anticipated impact and dissemination**: Key outputs from TRANSLATE (Guidelines on the management of FEP, EIP service adapted for LMIC, a prognostic model to predict TRS soon after FEP diagnosis, about 20 clinicians and postgraduates trained in EIP and implementation research, about six publications) will have a substantial academic, societal, and economic impact and will be disseminated widely. |
| **Scope of Protocol** | Work Package (WP) one  *Work Package Two will be described in a separate protocol* |
| **Study period** | September 2024- August 2025 |
| **Study objectives** | 1. To implement a culturally sensitive EIP service in Pakistan and Sri Lanka based on recent guidelines for managing FEP adopted for LMIC settings. **(WP 1 & WP 2)**   **This protocol is relevant to WP1**   2. *To evaluate the implementation of EIP in maintaining engagement with the services, achieving remission in FEP, and other relevant implementation outcomes.* ***(WP 2)*** 3. *To assemble a cohort of FEP within the EIP service and identify potential predicting factors of TRS at one-year follow-up* ***(WP 2)*** 4. *To develop a prognostic model for estimating an individual's risk of treatment resistance at one year and to undertake the validation of the model's predictive performance* ***(WP 2)*** 5. *To identify barriers and facilitators for the scale-up of the EIP at regional and international levels.* ***(WP 2)*** 6. *To build capacity for implementation research and service development for youth mental health in LMIC* ***(WP 3)*** |

**TRANSLATE Study flow**

# 1. BACKGROUND

## 1.1  Research Importance

First Episode Psychosis (FEP) typically refers to the first ever psychotic episode (schizophrenia and other psychotic disorders) in people who either have not received any antipsychotics or have used the medication for no longer than six weeks (Breitborde et al., 2009). Duration of Untreated Psychosis (DUP), the period between the onset of psychosis and initiating treatment, is 125 weeks in low-and middle-income countries (LMICs) compared to 62.5 weeks in high-income countries (HICs) (Lora et al., 2012). Even when treatment begins, it typically only addresses acute episodes. Almost 70% of people with psychosis do not receive regular care, resulting in a huge treatment gap (De Silva et al., 2014b). Untreated or partially treated FEP disrupts education, reduces long-term employment, causes a huge economic burden, and is associated with significantly higher mortality than the general population (Farooq et al., 2009) . About 10% of patients self-harm during long periods of untreated psychosis (Lora et al., 2012). Farooq et al. mapped the components of EIP services described in the included studies of the review against the seven standards in the National Institute for Health and Care Excellence (NICE) guidelines. This review provided a framework for the development of a service model for EIP and interventions that can be used to manage individuals with FEP in resource-poor settings (Farooq et al., 2024)

Almost one-third of people with schizophrenia (which constitutes about 70% of all FEP) develop treatment-resistant schizophrenia (TRS) which is defined as failure to respond to two sequential courses of antipsychotic treatment. Patients with TRS have higher unemployment rates, a worse quality of life, and poorer social and occupational functioning than those who respond to treatment (Kennedy et al., 2014). All schizophrenia treatment guidelines, including the NICE guidelines, recommend starting with clozapine (the only medication licensed for TRS) for TRS. However, treatment with clozapine is often delayed for about ten years globally (Wheeler, 2008) and is generally prescribed to less than 5% of patients who would benefit from the drug, even in high-income countries (Farooq & Taylor, 2011). Other guidelines recommend interventions such as cognitive behavioral therapy (CBT) are used even less optimally (Berry & Haddock, 2008). The lack of evidence-based treatment for FEP is a huge public health problem in LMICs, while the delayed use of Clozapine and other interventions for TRS is a global problem both in LMICs and HICs

## 1.2 Review of existing evidence

There is overwhelming evidence that deterioration occurs aggressively in the first 2 to 5 years of early psychosis, and it is crucial to intervene in this period. This has prompted the development of early intervention for psychosis (EIP) programs in HIC. EIP services provide treatment as soon as possible after the FEP diagnosis. Systematic reviews and meta-analyses have shown that EIP services result in significantly improved outcome (Fusar‐Poli et al., 2017; Mcdaid et al., 2016) , including a reduction in psychiatric hospitalisations, reduced symptom severity, and improved personal and social functioning, with a higher number of patients returning to employment or education (Correll et al., 2018; Pipkin, 2020). Furthermore, various settings and models of early intervention demonstrate improved outcomes (Correll et al., 2018; Fusar-Poli et al., 2017; Pipkin, 2020). Almost 90% of 1.8 billion young people aged 10 to 24 years - the highest risk period for early psychosis - live in LMIC countries (Gupta, 2014). Despite the limited evidence in a few countries, Farooq et al. found that low- and middle-income countries (LMIC) can establish and adapt the key elements of early intervention in psychosis (EIP) to settings with limited resources (Farooq et al., 2024).

A recent systematic review suggested that starting clozapine early in treatment could improve the outcome of patients with first-episode schizophrenia (Okhuijsen-Pfeifer et al., 2020). Specifically, Yoshimura et al. (2017) showed that earlier use of clozapine resulted in significantly higher response rates (81.6% versus 30.8%) compared to the delayed initiation (Yoshimura et al., 2017). A systematic review carried out by Farooq et al showed that lack of awareness and difficulty in identifying TRS in the early stages is a major problem resulting in long delays and underutilization of clozapine (Farooq et al., 2018).

## 1.3 Stakeholder, patient, and public engagement

TRANSLATE's research plan is based on our extensive consultation with service users and community members. These consultations led to the following improvement/changes in the research plan: greater involvement of traditional and spiritual healers in treatment pathways, assessment of physical health in all assessments, plans to involve educational institutions in campaigns to raise awareness about psychosis in young persons and about the EIP, involvement of teachers and educational psychologists in providing psychological support for young persons with FEP. This consultation also raised our awareness about the need for better cultural sensitivity in assessing female patients, involving traditional healers and local councilors in dissemination activities, and preparing all information materials in local languages

## 1.4 Work Packages

The research plan will consist of three interrelated work packages (WPs):

**WP-1 (0-7 months):** We will develop a model for EIP using the theory of change workshops, focus groups, in-depth interviews, and consensus-building workshops.

**This proposal is to obtain ethical approval for the WP -1. The ethical approval for the WP-2 will be obtained later.**

**WP-2 (7-52 months)**: The resulting model will be tested in a small pilot to refine procedures, measurements, and outcomes. We will establish EIP in six sites in Pakistan and Sri Lanka. We will recruit 670 people in the service and evaluate the clinical utility of EIP in terms of rates of engagement and remission at one-year follow-up, acceptability, feasibility, and costs to patients, and from the payer and provider perspective. All patients recruited for the EIP and started on antipsychotic treatment will form the cohort for developing and validating a clinical tool for predicting the risk of TRS. The potential predictors for TRS and occurrence of TRS will be measured at baseline, 1,3,6, and 12 months. A similar study will guide the development and internal validity we completed in two UK cohorts.

**WP-3 (0-60 months):** We will develop a consortium of researchers who will work with PhDs and postdocs to seek external funding to scale up EIP in other countries, build capacity and enhance the impact. The WP will run through the course of the study.

# 2 METHODS

## 2.1 Study Design

This study has an implementation design that aims to improve the adoption, appropriate adaptation, and delivery of EIP, building on existing evidence for its efficacy and effectiveness. For Work Package 1, we will be using stakeholder engagement and qualitative study design.

## 2.2 Study setting

TRANSLATE will be based in 4-5 districts in Pakistan and one in Sri Lanka. The study setting for the in-depth interviews will be the UK.

Pakistan's population is 231 million (median age 22.8 years). Each of the four Pakistan provinces will have a study site to ensure a good representation of urban and rural populations and all ethnic groups. The study sites will be in four or five major districts (population in millions in parenthesis): Districts Peshawar (2.34), Abbottabad (1.3) in Khyber Pakhtunkhwa (KP), Faisal Abad (2.32) in Punjab, Quetta in Baluchistan (1.16), and Hyderabad in Sind (1.73). All sites have agreed to support the research, and the heads of psychiatry departments at these sites will be the site collaborators. In Sri Lanka, TRANSLATE will be based in Galle district (population 1.1 million) at Karapitiya Teaching Hospital.

## 2.3 This protocol

This protocol specifically outlines the research activities of WP1. We will develop a separate protocol to describe the research activities related to WP2, guided by the outcomes and findings of WP1 (please refer to the study flow chart).

# 3. **WORK PACKAGE ONE** – STAKEHOLDER ENGAGEMENT AND QUALITATIVE STUDIES TO UNDERSTAND THE IMPLEMENTATION CONTEXT (objective 1)

- To discuss and develop consensus guidelines for the management of FEP.
- Three one-day workshops will involve a broader range of stakeholders from all six participating sites.
- An adopted Nominal Group Technique will be used.
- Draft the EIP services to be piloted.

**Workshops (TOC)**

**(2-5 months)**

- With key stakeholders from diverse backgrounds
- To outline a map of resources, processes, treatment strategies, long-term outcomes, and impact in managing FEP.
- Three one-day workshops and each will include up to 15 participants.
- Focus group discussions with service users, psychiatrists, primary care workers, and traditional healers.
- To explore the views on the EIP model and managing FEP
- Six to eight FGDs will take place with 6-8 participants per focus group.
- Four to five in-depth interviews with mental health professionals working in EIP services in the UK to elicit their views on adopting the EIS model for the LMIC setting
- Thematic analysis will be carried out and key themes will inform the EIP model and guideline development.

**WP1 Flowchart (0-7 months)**

**Qualitative study**

**(3-5months)**

- One psychiatrist from each of the six study sites will be trained.
- Act as a master trainer and clinical supervisor for the management of FEP and work with the primary care physicians.
- A clinical psychologist will provide training and supervision for psychological therapies.

**Cascade Training**

**(4-7 months)**

**Consensus building**

**(6-7months)**

## 3.1 Theory of Change (TOC) Workshops

Three Theory of Behaviour Change (ToC) workshops will be held with about 15 stakeholders: healthcare providers, service users, and health administrators. The study team will use its pre-existing professional networks to identify stakeholders. ToC is a participatory theory-driven approach to program, design, and implementation; it has been applied previously to inform complex intervention development in LMIC settings (De Silva et al., 2014a). The workshops will facilitate the exchange of knowledge among stakeholders, allowing for in-depth discussions on particular aspects of the TOC. We will encourage the participants to express their views while assessing the feasibility of adopting the suggested guidelines and EIP model in a resource-poor setting.

The research team includes early career researchers who are trained on the TOC concept. Through facilitated discussion, the participants will work together to outline a map of resources, processes, treatment strategies, long-term outcomes, and impact in managing FEP. TOC will describe causal pathways to achieve desired outcomes and impacts and help identify barriers and facilitators for implementation of the EIP model.

At the end of eachworkshop, the TOC map and key insights will be shared with the participants for their feedback and validation.

## 3.2 Qualitative study

## 3.2.1 Focus group discussion

We will conduct 6-8 focus groups (FG), in different study sites, with service users, health care professionals, health administrators, and traditional spiritual healers (TSH). This approach, designed to foster a collective conversation among a group of people, is valuable for examining different viewpoints in a manner that overcomes the limitations of a one-on-one interview. This effect occurs because of the group process, which prompts individuals to provide feedback on one another's experiences and perspectives (Carey & Asbury, 2016; Kitzinger, 2013), resulting in a deeper and better understanding.

We will conduct five or seven FGDs in Pakistan, including two or three FGDs with psychiatrists, one with TSHs, one or two with service users, and one with health administrators. In Sri Lanka, we will conduct two FGDs with the health care professionals and service users. The aim is to capture a broad range of perspectives on EIP and managing FEP.  However, the number of FGDs will be determined by the reaching the concept of theoretical saturation and theoretical data points. Each FGD will include 6-8 participants. FGD methods will collect the shared views, and opinions and will provide the foundation for developing the model and guidelines. Focus group discussions will be conducted by experienced qualitative researchers in the research team (moderator and note taker) using a topic guide. Each FGD will last approximately 60-90 minutes, will be recorded, with permission, and transcribed. Field notes will be written with each FGD. The analysis will be guided by the research questions and will be on-going throughout the fieldwork. It will involve an iterative approach, whereby themes will be identified and fed back into the data collection process. This method of moving between identifying themes and analysing data will facilitate a better understanding of the attitudes, practices, acceptability, and optimisation of the EIP services. The researchers who will collect and analyse the FGD data are fluent in the local language and are trained in qualitative research methods.

We will choose a convenient and familiar setting, such as a community health centre or clinic, for the discussion, taking into account the convenience of study participants. Key themes will then inform the model and guideline development to be taken forward in subsequent parts of the WP-1.

#### 3.2.1.1 Participant Recruitment for the FGD

We will select study participants from local healthcare facilities and Traditional and Spiritual Healers (TSH) who serve the area. The research team members have extensive experience in conducting similar research and have established strong connections with the community and stakeholders. We will use these connections to recruit suitable participants for the FGDs.

#### 3.2.1.2 Inclusion criteria

Service users: We will recruit participants aged between 18 and above who have a diagnosis of psychosis or schizophrenia and have experience using mental health services.

Healthcare professionals, health administrators, and traditional spiritual healers: We will select participants from the current research study's local area who manage and provide mental health services.

### 3.2.2 In-depth interviews

We plan to conduct four to five in-depth interviews with mental health professionals in the UK who have experience working in mental health services in LMIC and who are familiar with the environment and the context of LMIC and working with EIP services in the UK. We aim to elicit their views on adapting EIP services for implementation in LMICs. Mainly we intend to gather their views on EIP service models, components, training needs, facilitators, and barriers to implementation. They will be recruited using the professional networks. The interviews will be conducted by experienced qualitative researchers in the research team. The insight gained from these interviews will be vital in developing an appropriate EIS model for the LMIC context that can be scalable with existing mental health care delivery systems. We will conduct the interviews for 40 to 60 minutes duration, either online or face-to-face at Keele University or in a clinical setting depending on the participant's convenience. We will obtain informed consent in writing at the start of the interview, if face-to-face, or beforehand via an online form if participating virtually (e.g., through MS Teams). Verbal consent will be checked and recorded at the start of the activity.

### 3.2.3 Data collection

We developed semi-structured topic guides to help generate and steer the discussion during the FGD and in-depth interviews.( Appendix 1 & Appendix 10). The guides will undergo repeated modifications as the data collection and analysis advance as well as considering the views of the members of the Patient and Public Involvement and Engagement (PPIE) advisory group. We will conduct focus group discussions (FGDs) in the participants' local language. We will also translate the topic guides into the respective local languages.

### 3.2.4 Data analysis

We will translate and transcribe the audio recordings and field notes into English for the data analysis. Trained and experienced research assistants (RAs) will conduct the initial transcription and translation, preferably on the same day, under the supervision of qualitative experts in the research team to ensure accurate transcription and translation of the data and opinions of the participants. A second transcriber will double-check the transcripts. The qualitative study team lead will approve the final version We will import the interview transcripts into the qualitative data-analysis software QSR NVivo 12 for thematic analysis.

## 3.3 Consensus-building workshops

We will conduct three one-day consensus-building workshops to discuss and develop the consensus guidelines for the management of FEP that are being developed as part of THE HOPE study. These workshops will aim to establish consensus on the content, structure, and format of the guidelines for managing and implementing FEP in EIP. An adapted Nominal Group Technique (NGT) (Maguire et al., 2022; Mcmillan et al., 2016) for brainstorming including structured rounds of explanation, idea generation, discussion, and ranking will be used. These workshops will include all six participating sites and involve a broader range of stakeholders: clinical psychiatrists, primary health care workers, psychologists, research team members, and members from the PPIE advisory group identified through the existing networks of the TRANSLATE project team. There will be 10–14 participants, and it will take place in a convenient location for them.

### 3.3.2 Stakeholder Engagement Workshop 1

Before the first consensus workshop, participants will receive the adapted guidelines as a part of the HOPE study, findings of the qualitative studies, the proposed EIP model with the components and literature evidence (eg: findings from the systematic review on early intervention in psychosis in LMICs (Farooq et al., 2024). We will also give them an outline of the meeting and the expected outcomes. The participants will work together to co-produce a working version of the guidelines' content, structure, and format and the EIP service model.

Members of the research team will facilitate the workshop. With verbal approval, the discussion will be digitally recorded to produce an accurate summary of the workshop proceedings.

### 3.3.3 Stakeholder Engagement Workshop 2

Before Workshop 2, the participants will receive a draft version of the template from Workshop 1, along with an outline of the meeting and its intended outputs. Stakeholders will collaborate to review the recommendations proposed by the adopted guidelines and the proposed EIP service model.

Members of the research team will facilitate the workshop. With verbal approval, the discussion will be digitally recorded to produce an accurate summary of the workshop proceedings.

### 3.3.4 Stakeholder Engagement Workshop 3

Before Workshop 3, participants will get an outline of the meeting and its expected outcomes, as well as a draft version of the Workshop 2 template. During the workshop, stakeholders will work collaboratively to review and finalize the recommendations from the adapted guidelines, the EIP service model adapted for LMIC.

Members of the research team will facilitate the workshop. With verbal approval, the discussion will be digitally recorded to produce an accurate summary.

## 3.4 Cascade training

We will use a cascade model of training and task shifting, which we have successfully used in previous research (Farooq et al., 2023) . The approach would involve experts in EIP from the UK providing training and supervision to master trainers in study sites, who will then cascade this training to local mental health and primary care workers on guideline-based management of FEP. At each participating site, one psychiatrist will act as a master trainer for other health workers and clinical supervisors for FEP management. A training module will be formulated and we will use face-to-face teaching, case studies, and direct clinical supervision, the same methodology as the THEHOPE study (Farooq et al., 2023).The primary investigator, Prof. Saeed Farooq, will conduct two sessions per week using online and/or face-to-face supervision during his visits to the sites for clinical service supervision. The Midland Partnership NHS Foundation Trust (MPFT) has agreed to support two NHS consultant psychiatrists (one working in EIP and another in child and adolescent psychiatry for managing young-onset psychosis) to provide one session a month for online clinical supervision. A clinical psychologist from the Pakistan Association of Cognitive Therapy (PACT) (https://pact.com.pk/) with expertise in cognitive therapy in early psychosis will provide similar training and supervision for psychological therapies.

We will develop training modules to provide guidance and support for the trainers, ensuring a smooth training process. The quality assurance of all training will be carried out by the quality enhancement unit of the Khyber Medical University, which is duly certified by the

Higher Education Commission of Pakistan. KMU will offer certificates upon completion of training to further motivate healthcare staff to participate in the study.

## 3.5 Informed consent and ethical conduct

### 3.5.2 Participant information sheet

Each eligible participant will be provided with a Participation Information Sheet (PIS) (Annexure 2,3,4,5& 11) for focus group discussions and in-depth interviews. We will translate the PIS into respective languages depending on the participants' language. It contains information on the research's objectives, the topics for discussion, and any other significant aspects of the study, such as potential burdens, benefits, and risks. The researcher will read the PIS when participants may have low literacy levels or are illiterate. Before giving their consent (Annexure6,7,8,9 &12), potential participants will have the chance to ask questions or voice any concerns they may have about their participation. We will encourage them to read it at home and contact the researcher if they have any questions. Additionally, we will also repeat the patient information sheet before taking the consent to make sure participants have understood and retained the information and asked any questions they may have.

A written consent will be obtained by signing the consent form. If a participant is illiterate, we will take a mark or thumb imprint in place of a signature if they are unable to write or read. We will assign a witness who is unrelated to our research study and has no influence over the participant. He/she will witness the researcher's procedure of reading out the information sheet, clarifying the questions raised by the participant, and taking the thumbprint.

The researcher will highlight that participants have the freedom to withdraw from the study at any point, up until the publication of the study findings, without needing to provide a reason. The data gathered up to that time will be secured according to data management protocols and will be included in the final analysis, unless the participant specifically requests otherwise. Withdrawal requests are not permissible after publishing.

### 3.5.3 Protecting participants’ privacy and confidentiality

We will assure participants that we will treat all information as confidential and secure it on password-protected computers. Upon obtaining participants' consent, interviews will be promptly recorded and transcribed fully following the event. The data will be securely stored under the Data Protection Act (2018) and only the study team will have access to it. All participants, as well as other individuals mentioned by them, will receive pseudonyms to protect their identities. We will seek permission to use participants' quotes verbatim in our reports.

Due to the group setting of the focus group discussion, the process for ensuring confidentiality during these interactions differs from that of a one-on-one research contact, such as an interview. To maintain anonymity, the research team will specifically request participants to refrain from disclosing any information discussed during the focus group or workshop to anybody outside of the group. We will also notify them at the outset so they can make an informed decision on how much to share. We will retain any information a participant provides, such as their contributions to the group discussion or activity, if they choose to withdraw from the study. We will refrain from retaining any personally identifiable information.

Before the focus group discussions, the participants will be verbally informed, and it is also included in the participant information sheet that confidentiality will be broken if the researcher identifies any danger to the participants. if the research team believes that participants are at risk of harming themselves or others, the information will be shared with relevant health care professionals .We will fully train the researchers to handle such situations and provide regular clinical supervision, enabling them to offer support and guidance to the participants on how to contact their general practitioner.

### 3.5.4 Participant burden

When engaging in qualitative research, it is crucial to refrain from overburdening a participant unduly. While we anticipate no significant burden on participants, some may perceive the research team as requiring more time and effort than they can provide.

To minimize any potential strain associated with interviews, it is important to always schedule them at a time and location that is convenient for the participants, and to ensure that the duration of the interview is reasonable. In addition, we will ensure that researchers follow culturally appropriate safeguarding procedures to ensure the safety of both participants and researchers. The researcher will also be trained and supervised to be aware of the risks associated with individuals experiencing depression or anxiety and take the appropriate steps to ensure safety. Participants may be excluded from the study if they are considered too ill on the research day. The research team will observe the participants for any signs of anxiety, distress, etc.

## 3.6 Data Management

The collection of all data will strictly comply with the MRC Data Management Policy, Data Protection Act (2018), and General Data Protection Regulation (GDPR) (2018).

During fieldwork, the researcher will keep the audio device safely in their custody. Following the fieldwork, the recordings of focus groups and in-depth interviewswill be securely transferred to the transcriber, via a password-protected server.

The partner institutions will subsequently store them on a secure password-protected server, accessible only to the country's research team members. We will save the pseudonymized transcripts of interviews on a secure server at Khyber Medical University, our partner institution in Pakistan, and the Institute for Research and Development, Sri Lanka, the partner institution in Sri Lanka. The aforementioned institutions will electronically store a list of study participants' details and their assigned pseudonyms in a secure file space, separate from research data. We will transfer electronic transcripts from Pakistan and Sri Lanka to the UK via a secure drive. . Keele University will store these on a secure network, requiring two-factor authentication (2FA) for access Keele University's network will house a study database containing participant information from the in-depth interviews, which requires a login and password to access. We will establish a shared folder (via MS SharePoint) to facilitate the secure sharing of research materials among collaborating partners, as and when deemed appropriate. To maintain confidentiality and anonymity, we will restrict the sharing of personally identifiable information. A data flow chart has been developed, describing the movement of data flow across the partner institutions is developed and uploaded (Annexure 14)

Inside the School of Medicine, there are secure physical storage arrangements for hard copies in lockable filing cabinets. We will preserve hard copies of research data for a decade after the findings are published. We will shred any printed, hard-copy research data for verification. We will wipe clean electronic media after this archiving period (if applicable) and permanently destroy physical data in accordance with the institutions' SOPs.

## 3.7 Patient and Public Involvement and Engagement

The TRNASLATE research plan necessitates robust patient, public, caregiver, and community engagement, with the goal of involving communities, patients, and carers in all stages of research. The nationally recognised PPIE team at Keele will guide these activities. All project governance and management committees will consist of a service user and a community member. Service users and community organisations who are already collaborating with us on ongoing studies in Pakistan will assist in selecting these members. We will also form a lay advisory group. The group will consist of 8-10 members from both Pakistan and Sri Lanka, who represent a diverse range of patients, service users, carers, and the community. The advisory group will aim to meet once every two months and will oversee the extended PPIE and community engagement activities, link with the study steering group, and advise the research team on various research activities. At least half of the advisory group will consist of women, those with lived experience of mental illness, and young people.

## 3.8 Study Outcomes -WP 1

Key outputs of the WP-1 stage one will be:

1. Fully developed consensus guidelines on managing FEP
2. A proposed EIP service model to be piloted

## 3.9 **Ethical and Regulatory Considerations**

### 3.9.2 Research Ethics Committee (REC) review & reports

The study will receive ethics approval from the Khyber Medical University (KMU) Ethical Review Board Pakistan, the University of Ruhuna Sri Lanka, and the Keele University Ethical Review Panel before the start of the study.

### 3.9.3 Peer review

The study was peer-reviewed as part of the grant application process by the National Institute for Health and Care Research (NIHR). Fully developed study protocol including all work packages will be submitted to a peer-reviewed Open Access Journal.

### 3.9.4 Access to the study dataset

Only members of the study team at Khyber Medical University (KMU) Pakistan, the Institute for Research and Development (IRD) Sri Lanka, and Keele University UK will have access to the final dataset. The CI will receive and evaluate requests to access data from external parties. The CI and the Project Management Committee will assess each request individually.

**Annexures**

| Annexure 1 | Topic guide for FGDs |
| --- | --- |
| Annexure 2 | Patient information sheet for Patients |
| Annexure 3 | Patient information sheet for health care professionals |
| Annexure 4 | Patient information sheet for Health Administrators |
| Annexure 5 | Patient information sheet for Traditional Spiritual Healers |
| Annexure 6 | Consent Form for Patients |
| Annexure 7 | Consent Form for healthcare professionals |
| Annexure 8 | Consent Form for Health Administrators |
| Annexure 9 | Consent Form for Traditional Spiritual Healers |
| Annexure 10 | Topic guide for in-depth interview |
| Annexure 11 | Patient information sheet for Mental Health Professionals UK |
| Annexure 12 | Consent form for Mental Health professionals UK |
| Annexure 13 | Gantt Chart |
| Annexure 14 | Data Flow chart |

References

Berry, K., & Haddock, G. (2008). *The implementation of the NICE guidelines for schizophrenia: Barriers to the implementation of psychological interventions and recommendations for the future*. Wiley. 10.1348/147608308x329540

Breitborde, N. J. K., Srihari, V. H., & Woods, S. W. (2009). *Review of the operational definition for first‐episode psychosis*. Wiley. 10.1111/j.1751-7893.2009.00148.x

Carey, M. A., & Asbury, J. (2016). *Focus group research*. Routledge.

Correll, C. U., Galling, B., Pawar, A., Krivko, A., Bonetto, C., Ruggeri, M., Craig, T. J., Nordentoft, M., Srihari, V. H., & Guloksuz, S. (2018). Comparison of early intervention services vs treatment as usual for early-phase psychosis: a systematic review, meta-analysis, and meta-regression. *JAMA Psychiatry, 75*(6), 555-565.

De Silva, M. J., Breuer, E., Lee, L., Asher, L., Chowdhary, N., Lund, C., & Patel, V. (2014a). Theory of Change: a theory-driven approach to enhance the Medical Research Council's framework for complex interventions.10.1186/1745-6215-15-267

De Silva, M.,J., Lee, L., Fuhr, D. C., Rathod, S., Chisholm, D., Schellenberg, J., & Patel, V. (2014b). Estimating the coverage of mental health programmes: a systematic review. *International Journal of Epidemiology, 43*(2), 341-353. 10.1093/ije/dyt191

Farooq, S., Choudry, A., Cohen, D., Naeem, F., & Ayub, M. (2018). *Barriers to using clozapine in treatment-resistant schizophrenia: systematic review*. Royal College of Psychiatrists. 10.1192/bjb.2018.67

Farooq, S., Fonseka, N., Ali, M. W., Milner, A., Hamid, S., Sheikh, S., Khan, M. F., Azeemi, M. M., Ariyadasa, G., Khan, A. J., & Ayub, M. (2024). *Early Intervention in Psychosis and Management of First Episode Psychosis in Low- and Lower-Middle-Income Countries: A Systematic Review*. Oxford University Press (OUP). 10.1093/schbul/sbae025

Farooq, S., Large, M., Nielssen, O., & Waheed, W. (2009). *The relationship between the duration of untreated psychosis and outcome in low-and-middle income countries: A systematic review and meta analysis*. Elsevier BV. 10.1016/j.schres.2009.01.008

Farooq, S., Sheikh, S., Dikomitis, L., Haq, M. M. U., Khan, A. J., Sanauddin, N., Ali, M. W., Ali, J., Khan, M. F., Chaudhry, I., Husain, N., Gul, M., Irfan, M., Andrews, G., Kaistha, P., Shah, S. M. U., Azeemi, I., Hamid, S., Minhaz, A., . . . Lewis, M. (2023). *Traditional healers working with primary care and mental health for early intervention in psychosis in young persons: protocol for the feasibility cluster randomised controlled trial*. BMJ. 10.1136/bmjopen-2023-072471

Farooq, S., & Taylor, M. (2011). *Clozapine: dangerous orphan or neglected friend?*. Royal College of Psychiatrists. 10.1192/bjp.bp.110.088690

Fusar‐Poli, P., McGorry, P. D., & Kane, J. M. (2017). Improving outcomes of first‐episode psychosis: an overview. *World Psychiatry, 16*(3), 251-265.

Gupta, M. D. (2014). *The power of 1.8 billion: Adolescents, youth and the transformation of the future*. United Nations Population Fund.

Kennedy, J. L., Altar, C. A., Taylor, D. L., Degtiar, I., & Hornberger, J. C. (2014). The social and economic burden of treatment-resistant schizophrenia: a systematic literature review. *International Clinical Psychopharmacology, 29*(2), 63-76.

Kitzinger, J. (2013). Using focus groups to understand experiences of health and illness. *Understanding and using Health Experiences: Improving Patient Care, 1*, 49-59.

Lora, A., Kohn, R., Levav, I., McBain, R., Morris, J., & Saxena, S. (2012). Service availability and utilization and treatment gap for schizophrenic disorders: a survey in 50 low- and middle-income countries: Bulletin of the World Health Organization. *Bulletin of the World Health Organization, 90*(1), 47-54B. 10.2471/BLT.11.089284

Maguire, T., Garvey, L., Ryan, J., Olasoji, M., & Willets, G. (2022). *Using the Nominal Group Technique to determine a nursing framework for a forensic mental health service: A discussion paper*. Wiley. 10.1111/inm.13023

Mcdaid, D., Park, A., Iemmi, V., Adelaja, B., Knapp, M., & Mcdaid, D. (2016). *Growth in the use of early intervention for psychosis services: An opportunity to promote recovery amid concerns on health care sustainability.* ().

Mcmillan, S. S., King, M., & Tully, M. P. (2016). *How to use the nominal group and Delphi techniques*. Springer Science and Business Media LLC. 10.1007/s11096-016-0257-x

Okhuijsen-Pfeifer, C., Sterk, A. Y., Horn, I. M., Terstappen, J., Kahn, R. S., & Luykx, J. J. (2020). *Demographic and clinical features as predictors of clozapine response in patients with schizophrenia spectrum disorders: A systematic review and meta-analysis*. Elsevier BV. 10.1016/j.neubiorev.2020.01.017

Pipkin, A. (2020). *Evidence base for early intervention in psychosis services in rural areas: A critical review*. Wiley. 10.1111/eip.13019

Wheeler, A. (2008). *Treatment Pathway and Patterns of Clozapine Prescribing for Schizophrenia in New Zealand.* ().10. 13451aph.1K662

Yoshimura, B., Yada, Y., So, R., Takaki, M., & Yamada, N. (2017). *The critical treatment window of clozapine in treatment-resistant schizophrenia: Secondary analysis of an observational study*. Elsevier BV. 10.1016/j.psychres.2017.01.064
