# Supplementary material for: Translating Evidence for Early Intervention in Psychosis (TRANSLATE): Protocol for Evaluating the Implementation of Early Intervention in Psychosis in Low and Lower‐Middle Income Countries (LMICs)
Source: Early Interv Psychiatry. 2026 Oct 21;20(10):e70243. doi: 10.1111/eip.70243 (PMC13686379; doi:10.1111/eip.70243)
Supplement: Supplementary file 3 [file EIP-20-0-s001.docx]

**Additional file 2- Diagram for the TRANSLATE Work Packages**
